# 3D visualization of myocardial substrate using Delayed Enhancement MRI for pre-planning and guidance of ablation procedures of ventricular tachycardia

**DOI:** 10.1186/1532-429X-13-S1-O56

**Published:** 2011-02-02

**Authors:** Jose L Rubio-Guivernau, Esther Perez-David, Ángel Arenal, Javier Bermejo, Andrés Santos, María Jesús Ledesma-Carbayo

**Affiliations:** 1 Universidad Politécnica de Madrid & Ciber BBN, Madrid, Spain; 2 Hospital General Universitario Gregorio Marañón, Madrid, Spain

## Background

In patients with prior myocardial infarction, intra-scar surviving fibers may create conducting channels (CC) which are the substrate of most sustained monomorphic ventricular tachycardias (VT). These channels can be identified by endocardial voltage mapping. Recent studies show high correlation between information from endocardial voltage mapping and delayed-enhanced MRI (DE-MRI) images.

## Methods

DE-MRI and left ventricular electroanatomic voltage maps (CARTO®) were obtained in 18 patients with chronic myocardial infarction referred for VT ablation. DE-MRI studies were performed with a 1.5 T Unit (Philips Intera®) in standard views, 10 min after injecting 0.02 mmol/kg of gadolinium contrast (Omniscan®), with 3D T1-TFE acquisition.

**Figure 1:**
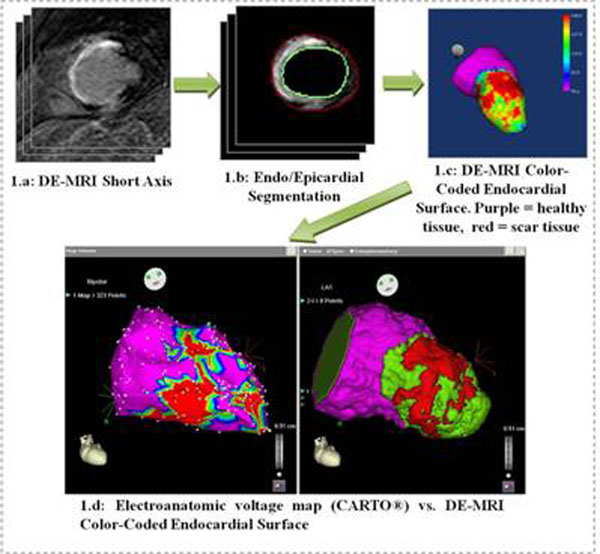


The 3D endocardial representation was computed off-line using proprietary software developed in the MATLAB environment (Mathworks). Starting with the short-axis view of the DE-MRI study (Figure 1.a), left ventricular endocardial/epicardial contours were manually defined (Figure 1.b) using QMass® MR 7.0 (MEDIS) and imported into our tool. Endocardial contours were used for generating the 3D shell on which we will project the color-coded information. The myocardial wall was then divided into 2 equal parts: subendocardium and subepicardium, and the averaged signal intensity (SI) of the inner half of the wall (subendocardial tissue) was projected onto the 3D endocardial shell reconstruction of the LV and color-coded for clear distinction of healthy (low SI), scar (high SI) and heterogeneous (medium SI) tissue (Fig. 1c). The resulting endocardial map was converted in a three region segmented volume to be integrated (Fig. 1d) into CARTO® navigation system using the CARTO MERGE tool (Fig. 1d). The feasibility of using the proposed visualization for guidance of ablation procedures was therefore tested.

## Results

The resulting color-coded endocardial shell was assessed by a cardiac electrophysiologist for comparison with electroanatomic voltage maps (Fig 1d). A CC was defined as a corridor of heterogeneous tissue with lower SI than surrounding scar tissue. For all of the 26 CC identified on the voltage maps for the 18 patients, similar (in location and orientation) channels were detected using the visualization generated from DE-MRI data alone. The use of the proposed visualization in the guidance of the ablation procedures was proved to be feasible and of interesting potential value.

## Conclusions

Given the agreement with electroanatomic voltage maps for identification of CC inside the scar tissue, we conclude that the proposed color-coded endocardial shell might be a useful tool for non-invasive location of such channels and for both pre-planning and guidance of ablation procedures.

